# MIPE: A metagenome-based community structure explorer and SSU primer evaluation tool

**DOI:** 10.1371/journal.pone.0174609

**Published:** 2017-03-28

**Authors:** Bin Zou, JieFu Li, Quan Zhou, Zhe-Xue Quan

**Affiliations:** Department of Microbiology and Microbial Engineering, School of Life Sciences, Fudan University, Shanghai, People’s Republic of China; Oklahoma State University, UNITED STATES

## Abstract

An understanding of microbial community structure is an important issue in the field of molecular ecology. The traditional molecular method involves amplification of small subunit ribosomal RNA (SSU rRNA) genes by polymerase chain reaction (PCR). However, PCR-based amplicon approaches are affected by primer bias and chimeras. With the development of high-throughput sequencing technology, unbiased SSU rRNA gene sequences can be mined from shotgun sequencing-based metagenomic or metatranscriptomic datasets to obtain a reflection of the microbial community structure in specific types of environment and to evaluate SSU primers. However, the use of short reads obtained through next-generation sequencing for primer evaluation has not been well resolved. The software MIPE (MIcrobiota metagenome Primer Explorer) was developed to adapt numerous short reads from metagenomes and metatranscriptomes. Using metagenomic or metatranscriptomic datasets as input, MIPE extracts and aligns rRNA to reveal detailed information on microbial composition and evaluate SSU rRNA primers. A mock dataset, a real Metagenomics Rapid Annotation using Subsystem Technology (MG-RAST) test dataset, two PrimerProspector test datasets and a real metatranscriptomic dataset were used to validate MIPE. The software calls Mothur (v1.33.3) and the SILVA database (v119) for the alignment and classification of rRNA genes from a metagenome or metatranscriptome. MIPE can effectively extract shotgun rRNA reads from a metagenome or metatranscriptome and is capable of classifying these sequences and exhibiting sensitivity to different SSU rRNA PCR primers. Therefore, MIPE can be used to guide primer design for specific environmental samples.

## Introduction

The elucidation of microbial community structure and diversity is an important issue in the field of molecular ecology. The traditional method is based on PCR, and requires the use of primers that specifically targets SSU genes to characterize a community [1]. The SSU rRNA genes, namely the 16S rRNA gene in Bacteria and Archaea and the 18S rRNA gene in Eukarya, have been widely used in microbial phylogeny since Carl Woese introduced the three-domain system [2,3]. This method has greatly expanded our understanding of microbial diversity and has led to the establishment of some public databases such as National Center for Biotechnology Information (NCBI) GenBank, SILVA, Ribosomal Database Project (RDP) and Greengenes [4–8]. Some studies and tools, such as TestProbe [8] and probeBase [9], have evaluated and improved these universal PCR primers based on these datasets. However, the accuracy of PCR approaches is reduced due to primer bias and chimeras, and these PCR-based databases and primer evaluation tools overestimate primer coverage [10].

The advent of high-throughput sequencing has given rise to a number of shotgun sequencing-based metagenomes and metatranscriptomes [11], and many related datasets have been accumulated. Consequently, some specific websites have started storing metagenomic data, such as the databases CAMERA, iMicrobe, and European Bioinformatics Institute (EBI) Metagenomics [12–14]. Because metagenomic and metatranscriptomic sequences are generated without PCR-based amplification, the rRNA sequences in these datasets are used for the microbial population analysis [15,16]. However, PCR amplicon sequencing is not out of date. Different from shotgun sequencing, PCR amplicon sequencing is economical, fast and able to be well aligned to analyze in standard workflows [17]. Several software packages have been developed, such as Mothur [18], Usearch [19] and QIIME [20]. Moreover, in ecology, a large number of environmental samples need to be analyzed in parallel to determine the contribution of environmental parameters to microbial populations, so PCR approaches are still widely used. We previously used different metagenome datasets to evaluate universal primers for the bacterial 16S rRNA gene and found that primer evaluation based on the RDP database overrated the coverage achieved with the primers [10].

Moreover, both primer coverage and PCR efficiency are important for the analysis of specific environmental samples. Increasing primer degeneracy would decrease the efficiency of PCR amplification and specific environments may require the use of specific primers. For example, when amplifying the bacterial 16S rRNA gene from gut samples, researchers mixed another primer with the widely used 16S rRNA primer 27F because the primer 27F cannot cover Bifidobacteria, which is dominant in gut samples [21]. However, 27F is broadly used in analysis of various environmental samples because the content of Bifidobacteria is relatively low in most water and soil samples. Therefore, the development of a software program for primer evaluation based on metagenome or metatranscriptome data from specific environmental samples is necessary.

Although some programs (SSUsearch, EMIRGE, MG-RAST API) [16,22,23] and websites (IMG, MG-RAST) [15,24] have been developed for the metagenomic microbial population analysis, no pipelines are currently available for primer evaluation. TestProbe [8] and probeBase [9] offer primer evaluation in their websites but rely on the PCR-based SILVA and RDP databases. Some programs, such as PrimerProspector [25] and DegePrime [26], evaluate and develop primers for taxonomic classification, but these programs cannot be used for metagenome sequence datasets and do not include rRNA extraction and global alignment processes, which are necessary for rejecting incorrect primer binding sites in shotgun reads. The tool De-MetaST [27] is available for metagenome datasets but was designed to provide *in silico* amplicons generated by user-defined degenerate primers found within a user-defined nucleotide database. Therefore, this software only provides information for the covered but not the uncovered parts and cannot be used for the evaluation of primer coverage or for primer modification. Furthermore, this software is not suitable for rRNA primers because it utilizes BLASTx (Basic Local Alignment Search Tool searching protein databases using a translated nucleotide query) for the classification.

To fill in the gap created by software programs for primer evaluation and the microbial population analysis based on shotgun metagenomes, we optimized our automated pipeline, which was established previously [10,28] and integrated it into the software MIPE (MIcrobiota metagenome Primer Explorer). MIPE extracts and classifies rRNA from a metagenome or metatranscriptome to provide information regarding community structure and allow the evaluation of different SSU PCR primers for different taxonomic groups. MIPE calls Mothur (v1.33.3) and the SILVA database for the identification of rRNA and the alignment of these sequences, which allows the identification of primer binding sites. The users only need to input the appropriate metagenome dataset and primer lists. The program automatically generates the output, including information regarding primer evaluation, rRNA sequence and taxonomy. This software also supports a modified process suitable for the same analysis based on a metatranscriptome, in this process, considerable rRNA reads would be extracted for the analysis of the primers. In the study described in this manuscript, MIPE was used to extract SSU sequences from a mock dataset and an MG-RAST metagenome dataset, and was used to evaluate the primer pair F515-R806 utilizing the standard SILVA database, the CAMERA metagenomic dataset and a metatranscriptomic dataset. The results were compared with those obtained using MG-RAST [15] and PrimerProspector [25].

## Materials and methods

### Design principles

**Fig 1:**
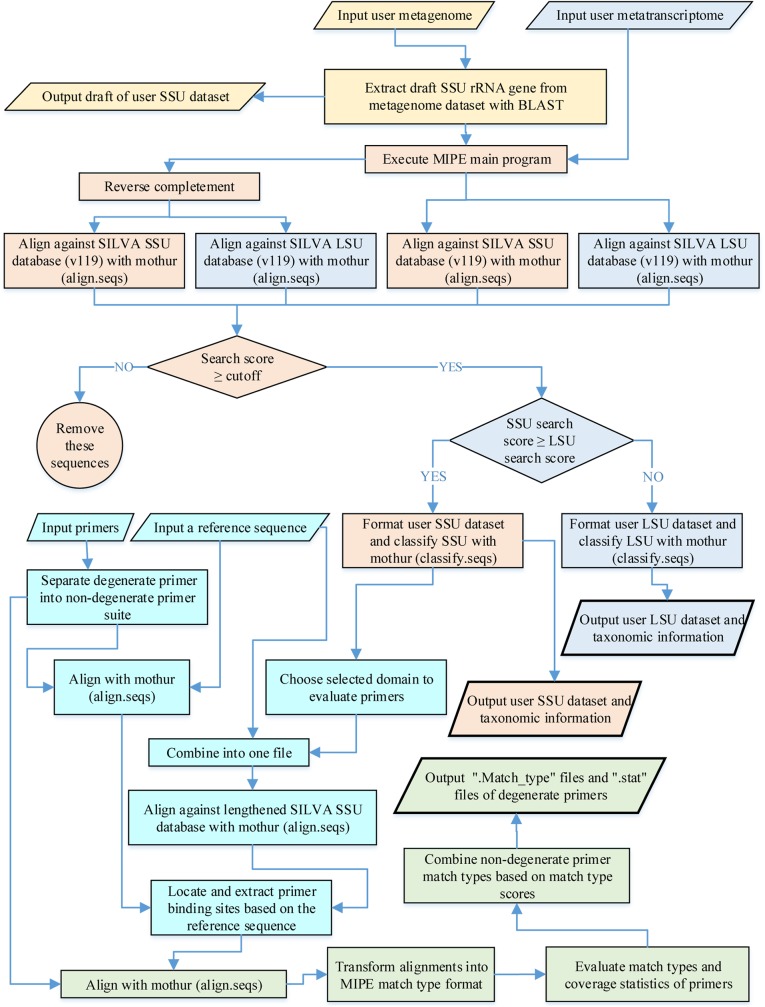
Work flow of MIPE. The part shown with a yellow background is the preprocessing stage of MIPE. The orange background details Stage I of the main program of MIPE, specifically sequence screening and taxonomy of a metagenome and a metatranscriptome, and the blue background describes steps used for metatranscriptomic sequence screening and taxonomy. The lake blue background details the steps associated with appending a reference sequence, locating and extracting primer binding sites, which form part of Stage I in the MIPE main program. The green background shows Stage II in the MIPE main program.

MIPE contains three perl scripts and depends on Mothur (v1.33.3) and BLAST (v2.2.26 or higher). MIPE can run under the Linux operating system only and can be downloaded at https://github.com/zoubinok/MIPE.git. MIPE uses private or public shotgun metagenomic or metatranscriptomic rRNA sequences submitted by the user to analyze community structure and SSU primer coverage. It calls Mothur (v1.33.3) and the SILVA database to align and classify rRNA, and a reference sequence is introduced to lock and mark primer binding sites. The reference sequence is separately aligned against the user-defined sequences and primers. Based on the reference sequence location, primer binding sites are extracted with three to five additional bases at both ends to avoid base slips resulting from multiple sequence alignment. The primers are then re-aligned against the primer binding sites obtained from the user-defined sequences to improve the alignment quality, and the sequences that are poorly aligned in this region are deleted. The workflow for MIPE is shown in Fig 1. MIPE can also be used to evaluate large subunit ribosomal RNA (LSU rRNA) primers only if the users replace the SSU reference sequence, the SILVA SSU database and the SSU primers with the LSU reference sequence, the SILVA LSU database (https://www.arb-silva.de/fileadmin/silva_databases/release_119/Exports/SILVA_119_LSURef_tax_silva_full_align_trunc.fasta.gz) and LSU primers, respectively.

### Data preprocessing

MIPE consists of two parts, namely dataset preprocessing and the main program. Dataset preprocessing is needed for metagenome datasets because only approximately 0.2% of the sequences in metagenome datasets are related to SSU rRNA genes [29,30]. To obtain SSU rRNA gene-like sequences in metagenome datasets, 71 representative sequences (45 bacterial, 17 archaeal and 9 eukaryotic sequences) were obtained by clustering the SILVA SSU database (v102) with Usearch (v5.2.32) [19] at a sequence identity level of 75%. These representative sequences are then used as queries in a BLASTn search against the user-defined sequences (default parameters: db alignments per query was 65535). A draft of the user-defined SSU dataset is then built to evaluate different primer sets. Because some hidden Markov model (HMM)-based tools can be used to replace this step, this step was not included in the main program [16,31,32]. For the analysis of metatranscriptome and amplified rRNA gene sequences, MIPE skips this step and runs the main program directly because rRNA gene sequences constitute a large part of these datasets. However, some rRNA sequences in some metatranscriptomic samples are removed for mRNA enrichment; in these cases, using the metagenome workflow would be a wise choice and these rRNA sequences cannot be used to reflect the community. In addition, for other special cases, such as cases with large insertions in SSU sequences, which would decrease alignment accuracy, it would prove beneficial to perform specific preprocessing with other methods before using MIPE [33]. But MIPE uses short reads from shotgun sequencing, so most large insertions would be excluded because of poor alignments in MIPE and then primers can be well evaluated, as well.

### MIPE main program

#### Stage I

This stage aims to classify sequences and extract primer binding sites. The detailed parameters are listed in the program.

The first step is the sequence screening and taxonomy of a metagenome. Based on the sequencing approach, genes may be reverse-complementary sequenced, which may have negative effects on the taxonomy and reduce the accuracy of the primer evaluation. To avoid this problem, each sequence and its reverse-complement sequence are aligned against the SILVA SSU database by calling “align.seqs” in Mothur (v1.33.3) [18]. The correct sequence trend is based on the comparison of two search scores that characterize the similarity of the candidate sequence with the reference database. If both values are lower than the cutoff search score which can be modified by the users, although a value of 30 is recommended, the sequence is not considered part of the SSU rRNA gene. Then, MIPE calls Mothur to classify (“classify.seqs”) the selected SSU rRNA gene sequences. If the sequence is classified into Bacteria, Archaea, or Eukarya and the bootstrap value is not less than the threshold cutoff (Mothur declares a minimum cutoff of 60, and 50 is also used for sequences shorter than 250 bp [28,34]), the sequence passes through to the next analysis.

Alternatively, the first step can be the sequence screening and taxonomy of a metatranscriptome. For metatranscriptome datasets, LSU rRNA is also considered because of its high content in the metatranscriptome [28]. Therefore, LSU is extracted in the same manner as SSU, and four search scores are acquired to determine the sequence attributions. The extracted LSU is also used for taxonomy and community analysis with the SILVA LSU database (v119) [8]. The subsequent metatranscriptomic SSU rRNA analysis is the same as that used for metagenomic datasets.

Appending a reference sequence, locating and extracting primer binding sites then follow in order. A reference sequence is then introduced to identify primer binding sites with MIPE. The default reference sequences are the standard full-length 16S rRNA gene sequence of *Escherichia coli* (GenBank accession number: J01695) and the corresponding sequence of *Methanomethylovorans hollandica* (GenBank accession number: NC_019977) and the 18S rRNA gene sequence of *Saccharomyces cerevisiae* (GenBank accession number: NR_132213.1). The user-defined SSU sequences and the reference sequence are combined and aligned against the aligned the SILVA SSU database (v119) using the multiple-sequence aligner “align.seqs” in Mothur (v1.33.3) so that every base is given a unique position on the reference sequence. Users would make a list of degenerate primers as input and make sure both their degenerate and non-degenerate formats have right primer binding sites on the reference sequence. Degenerate primers are split into non-degenerate primers with MIPE, and these non-degenerate primers are aligned against the reference sequence using the same tool. The reference sequence, which is used as a marker, helps extract the primer binding sites in the user-defined SSU sequences.

The SILVA SSU database (v119) that is used with MIPE was downloaded from the Mothur modified Recreated SEED database (http://www.mothur.org/w/images/5/56/Silva.seed_v119.tgz). This database does not have the primer binding regions 8F and 1492R because most bacterial sequences were amplified using this primer pair. To address this shortage, all of the sequences were aligned against an extended SILVA database that was prepared in house. To build the extended SILVA database, the primer 8F was attached to the 5’ region of every sequence in the SILVA SSU database (v119) and the reverse complement of the primer 1492R was attached to the 3’ region of every sequence. A perl script (SILVA_ENLONG.pl) was provided as a part of MIPE in github.

#### Stage II

This stage aims to analyze and output the results from primer matching. The Mothur command “align.seqs” is called for the alignment of every extracted primer binding site from the user-defined SSU sequences against the non-degenerate primers to rectify any base slips caused by multiple sequence alignment. Four types of mismatches in the primer binding sites, namely substitution, insertion, deletion and missing fragment, are marked with different signals based on the results of this alignment.

**Fig 2:**
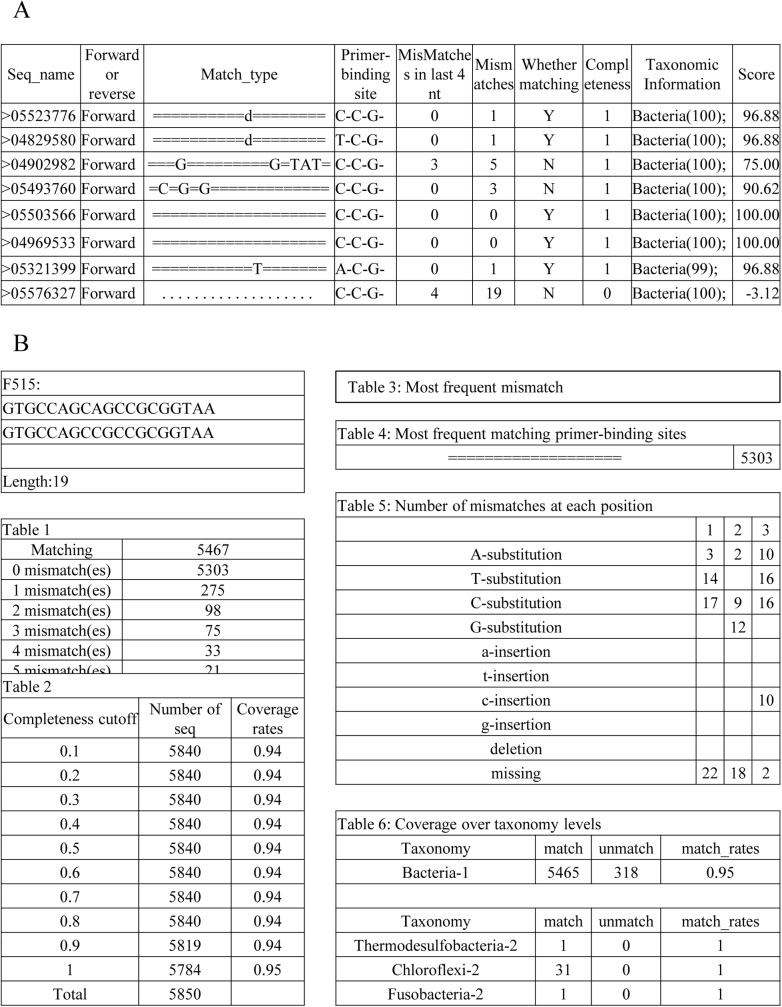
Format of MIPE output. (A) Match type file, “.Match_type”. The file contains each match type for the sequence, the sequence binding sites for the primers, the mismatch base numbers in total, the mismatch base numbers in last 4 bases, the matched degree, the completeness, the taxonomy information and the score. (B) Statistics file, “.Stat”. The file presents a summary of the primer evaluation, and all tables result from the analysis of the “Match_type” files. The file contains six tables: the match and mismatch sequence count, the distribution of completeness, the most frequent mismatch type, the most common match type, the count of each mismatch in every position, and the coverage over taxonomy levels. Rare biospheres can be found in “Table 6” of the statistics file and sequences whose completeness were less than one were not counted in this table. For more details, please see S3 Table.

For each non-degenerate primer, MIPE outputs two files, a match type file (“.Match_type”) and a statistics file (“.Stat”). The “.Match_type” file includes details from each sequence evaluation. Five match types, namely match, substitution, insertion, deletion and missing fragment, are expressed by the MIPE match type formats ‘ = ‘, ‘A(TCG)’, ‘a(tcg)’, ‘d’ and ‘.’, respectively, to facilitate the statistical analysis which involves each match type for the sequence, the binding sites of the primers, the mismatch base numbers in total, the mismatch base numbers in last 4 bases, the matched degree and the taxonomy information. A single internal primer-template mismatch can greatly decrease PCR efficiency, particularly if the mismatch occurs at the last three to four positions; thus, the degree of matching is defined as “no mismatching” or “only one mismatch that is not found in the last four positions near the 3’ end” [35,36]. The match types of each non-degenerate primer separated from each degenerate primer are integrated into “.Match_type” to evaluate degenerate primers using the best match type, which is based on the score provided in the “.Match_type_tmp” files. The format of “.Match_type” is shown in Fig 2A.

The “.Stat” file provides a summary of the primer evaluation. All of the results were generated by the analysis program using the “Match_type” files. The “.Stat” file contains six tables: the match and mismatch sequence count, the distribution of completeness, the most frequent mismatch type, the most common match type, the count of each mismatch in every position, and the coverage over taxonomy levels. To reflect the mismatch of any missing information, we introduced the variable “completeness” for each primer binding site. If the primer binding site of a fragment was too marginal to cover all of the bases of a primer or was poorly aligned, this site would not be counted when evaluating the coverage of a certain primer. The format of the “.Stat” file is shown in Fig 2B.

### Testing the dataset and demonstration

#### Comparison of sequence taxonomies

A 13-organism genomic mock dataset was simulated using MetaSim (version 0.9.1; [37]) with a sequence length of 400 bp for each organism, 6X coverage and an exact error model to confirm the accuracy of the taxonomy obtained by Stage I in the MIPE main program. The 13 organisms consisted of two Archaea, nine Bacteria and two Eukarya based on the MetaSimHC database [38] and their GI numbers were 159184118, 159185562, 75906225, 11497621, 42521650, 121612099, 15893298, 116510843, 30248031, 32141095, 24473558, 330443681 and 453231596. MIPE extracted SSU sequences from the mock dataset (search score cutoff: 10; bootstrap cutoff: 80), and a goodness-of-fit analysis based on Pearson's Chi-squared test at the genus level was executed with R (version 3.1.2; http://www.R-project.org/).

A metagenome sequence dataset from an activated sludge sample (MG-RAST ID: 4467420.3) [39] was also downloaded from the MG-RAST website (http://metagenomics.anl.gov/) [15] to evaluate the effects of different methods and databases. This sample was analyzed with the MG-RAST built-in SSU databases, through the MG-RAST pipeline (Metagenomes: 4467420.3; Annotation Sources: SSU; Max. e-Value Cutoff: 1e-5; Min. % Identity Cutoff: 60%; Min. Alignment Length Cutoff: 15). The downloaded raw data (FASTA format) were analyzed with MIPE (search score cutoff: 30; bootstrap cutoff: 50). Linear correlations were calculated at the phylum and class levels of Archaea and Bacteria. Taxa whose abundances are greater than 100 reads in either the SILVA SSU (v119) or MG-RAST built-in SSU databases were listed separately and the others were summed into one item. Due to differences in taxonomy information for Eukaryota between the SILVA SSU (v119) and MG-RAST built-in SSU databases, the test for Eukarya was not executed.

#### Primer evaluation

To compare the primer evaluation results obtained by MIPE and PrimerProspector [25], the “SILVA test set” and “Metagenome test set” were used. The “SILVA test set” was used with the example pipeline of PrimerProspector, derived from the SILVA SSU database (v104) and filtered at 97% sequence identity with Uclust [19]. The “Metagenome test set” was selected from the CAMERA website (release v.1.3.2.30; http://camera.calit2.net/) based on our previous work in 2012 [10,14]. The primer set F515-R806 [25,40,41] (F515: 5’-GTGCCAGC(A/C)GCCGCGGTAA-3’; R806: 5’-GGACTACC(A/C/G)GGGTATCTAAT-3’), which is designed to be universal for nearly all bacterial and archaeal taxa, is widely used in high-throughput sequencing to amplify V4 region of 16S SSU rRNA. We used this primer set for the evaluation. It was also evaluated with the “SILVA test dataset” via PrimerProspector. The MIPE parameters were a search score cutoff of 30, a bootstrap cutoff of 80, the inclusion of Bacteria and other default parameters. The scripts “analyze_primers.py” and “taxa_coverage.py” with default parameters in PrimerProspector were used and the PrimerProspector results were transformed into the MIPE match type format for the comparison. Because the primers F515-R806 are bacterial primers, only bacterial SSU sequences classified by MIPE were compared. In PrimerProspector, a primer binding site with an overall weighted score of at most 1.00 was regarded as a matched site. The phylum-level coverage data presented in the MIPE output “Table 6: Coverage over taxonomy levels” from a “.Stat” file were used for comparison.

The metatranscriptomic dataset SRX155355 (Short Read Archive (SRA) accession number: SRX155355), which is based on our previous work in 2014 [28] was executed using the metatranscriptome section of MIPE. All sequence reads containing “N” were discarded and sequences longer than 400 bp were used. The sequences were checked for chimeric artifacts using the “chimera.uchime” in Mothur (v1.33.3), and the passed sequences were processed with MIPE metatranscriptome (search score cutoff: 20; bootstrap cutoff: 80).

## Results and discussion

### Extraction and taxonomic identification of SSU sequences

Running time of MIPE (CPU time) was tested using the 13-organism genomic mock dataset containing 1000000 sequences. MIPE was run on Ubuntu 14.04.1 (2×2.40GHz Quad-core Xeon, 64GB of RAM) but in the single-threaded mode. The dataset preprocessing (MIPE_pre_program.pl) took 3 minutes and extracted 2389 sequences. MIPE main program (MIPE_main_program.pl) took 5 minutes to deal with these 2389 sequences.

The SSU sequences in the 13-organism genomic mock dataset were extracted and classified by MIPE. A goodness-of-fit analysis based on Pearson's Chi-squared test (chisq.test in R and the R code was in S1 Table) at the genus level showed no significant difference between the original percentage and the MIPE-determined percentage (p value > 0.05; S1 Table) indicating that MIPE can extract SSU sequences from an unbiased mock dataset.

**Fig 3:**
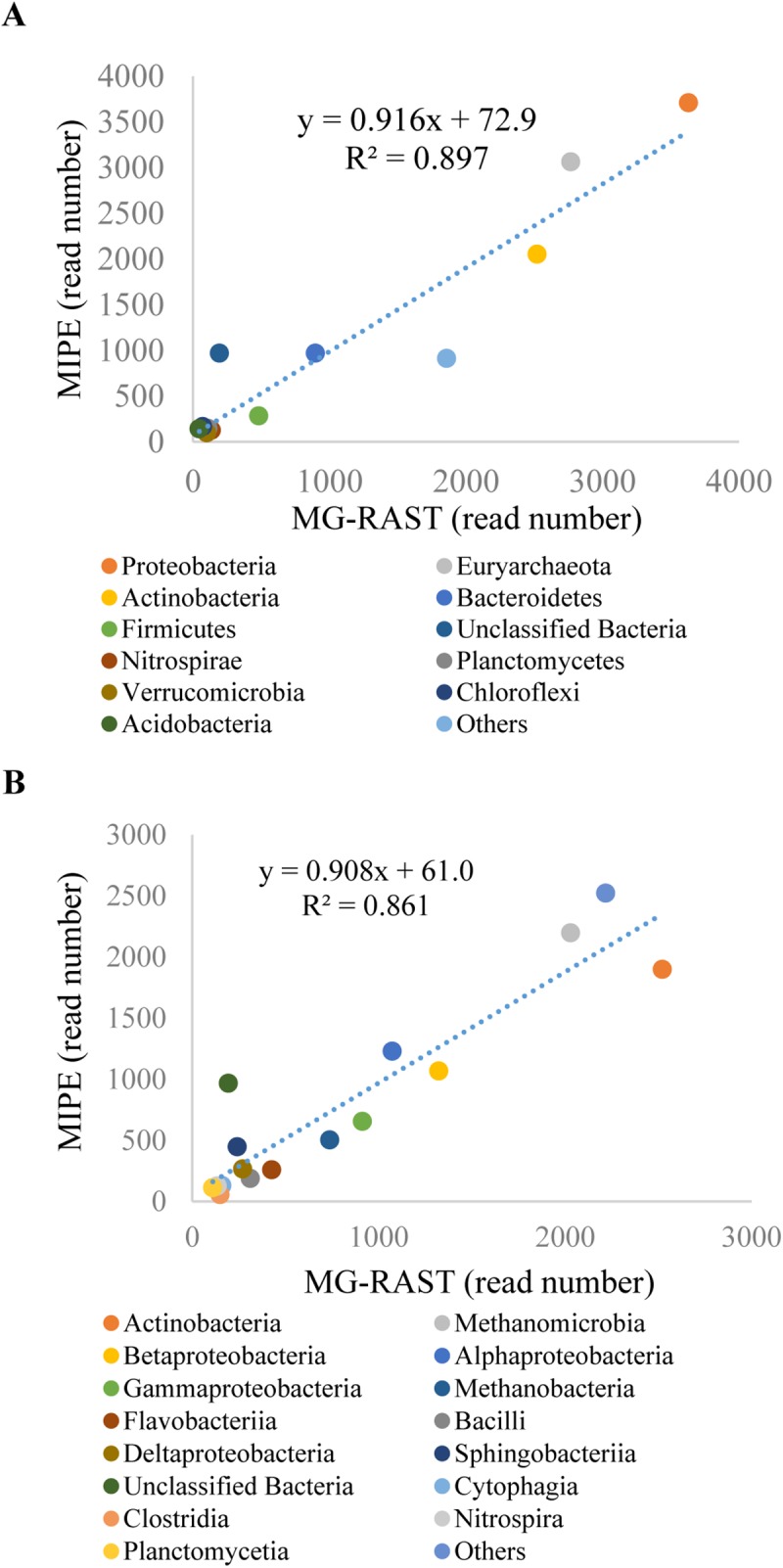
Linear correlation-based comparison of MG-RAST and MIPE. Archaeal and bacterial read numbers used for linear correlation at the (A) phylum and (B) class levels. Taxa whose abundances are greater than 100 reads in either SILVA SSU (v119) or MG-RAST built-in SSU databases are listed separately, and the others are summed into one item.

The activated sludge sample from MG-RAST dataset 4467420.3 (16663946 reads) was evaluated. Krnoa charts [42] of SSU sequences show the MIPE results, revealing the relative abundance distribution of microbes at different taxonomic levels (S1 and S2 Files). Based on the two results, the main clusters of Bacteria and Archaea are similar. A low content was detected with MIPE for the Eukarya cluster because the taxonomic database SILVA SSU (v119) used for the MIPE analysis does not cover fish, such as *Danio*, the dominant Eukaryote in this dataset. MG-RAST is broadly used for microbial analysis of shotgun metagenome, which identified rRNA sequences through an initial BLAT search [15]. But MG-RAST built-in SSU database is not available for us and it cannot be replaced. Furthermore, MG-RAST only reports the relative abundance and we cannot get the taxon of every sequence. Thus, we used linear correlation-based comparison instead. Fig 3 clearly shows the significant linear correlation of Archaea and Bacteria read numbers at the phylum (R^2^ = 0.897, P = 2.99 e-06) and class level (R^2^ = 0.861, P = 2.27 e-07) between the results obtained using the two methods. In conclusion, MIPE can efficiently extract SSU rRNA gene sequences from metagenome datasets and accurately identify the taxonomy of each sequence.

The metatranscriptomic dataset SRX155355 contains 21,035 sequences prior to processing by MIPE. MIPE extracted 10,885 SSU sequences and 7452 LSU sequences. Thus, rRNA sequences accounted for 87.2% of the total sequences. Considering that the whole amount of rRNA genes in a metagenomic dataset is approximately 0.2%, metatranscriptomic datasets provide considerable sequences.

MIPE is flexible because it is a local pipeline to which different SSU or other gene databases can be applied for the analysis of data from different environments because different environments consist of different communities and it is better to select primers for specific environments.

### Validation of the primer evaluation process

The primer set F515-R806 were evaluated using the “SILVA test set” and “Metagenome test set” with MIPE and PrimerProspector [25]. Because PrimerProspector was not designed for metagenomes and cannot perform global alignments or classify metagenome sequences using its primer evaluation modules (analyze_primers.py and taxa_coverage.py), we had to input the SSU rRNA gene sequence and taxonomy files for the comparison.

**Fig 4:**
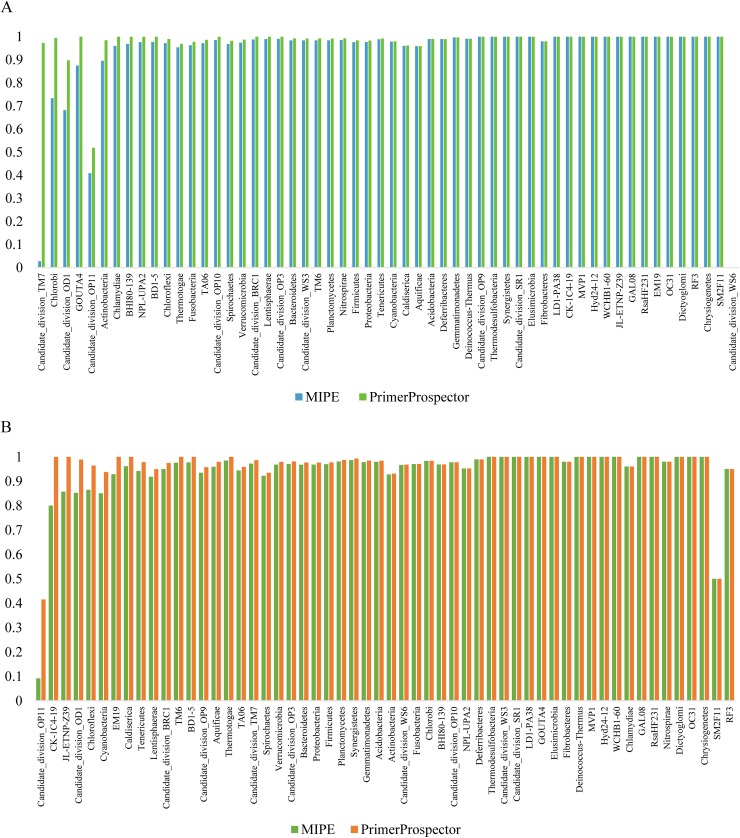
**Coverage of the “SILVA test set” with the primers F515 (A) and R806 (B) obtained using MIPE and PrimerProspector.** The SILVA test set sequences were derived from the SILVA SSU database (v104) and filtered at 97% sequence identity with Uclust. The y-axes represent the percent of coverage.

The coverage of most phyla by the primers F515 and R806, as evaluated by MIPE, basically agreed with the results obtained by PrimerProspector, presenting a 0%-to-10% difference (Fig 4) in the “SILVA test set. The different coverages obtained by these two programs were due to different penalty rules. But, in fact, they had the same match type. In other words, there were no differences between two results except penalty rules and penalty rules can get changed in scripts. The details are shown in sheets “F515_annotation_of_difference” and “R806_annotation_of_difference” of S2 Table. Other sheets of S2 Table original match type files and statistics files.

In the analysis of the “Metagenome test set”, PrimerProspector found one primer binding site in every sequence for primer F515 or R806, which means that PrimerProspector identified incorrect primer binding sites, because it only performed local alignments and did not anchor the primer binding sites, even if it was an incorrect site. MIPE avoids this problem through the use of a global alignment and the anchoring of primer binding sites. Because the results obtained by PrimerProspector included incorrect information for uncovered sections and cannot be used for primer evaluations and metagenome modifications, just like De-MetaST [27], we can only compare the match types from these two scripts case by case (Sheet: F515_annotation_of_difference and F806_annotation_of_difference in S3 Table).

The metatranscriptomic dataset SRX155355 contained 3915 bacterial sequences which had primer binding sites for F515 and 2081 bacterial sequences had primer binding sites for R806. Thus, primer binding sites for F515 and R806 accounted for 18.6% and 9.9% of the total sequences, respectively (see S4 Table). Metatranscriptomic datasets provided considerable sequences for testing primers.

## Conclusions

A priori knowledge is important when selecting and developing primers for the microbial population analysis. MIPE is a pipeline that combines automated SSU primer evaluation with rRNA gene extraction and classification for the analysis of shotgun metagenomic and metatranscriptomic datasets, which are becoming increasingly available. Furthermore, "missed" microorganisms from previous studies can also be discovered with newly designed primers based on mismatched types. In the future, MIPE will be able to address other marker genes to offer users a more powerful analysis tool for discovering the features of functional microbial communities, and an SSU rRNA gene database based on metagenomic or metatranscriptomic sequences can be established based on results obtained using the MIPE pipeline.

## Supporting information

S1 Table MIPE results obtained using a mock dataset and the R code. (XLS)

S2 Table Details of the output obtained from the MIPE and PrimerProspector analyses of the “SILVA test set” with the primers F515 and R806. (XLSX)

S3 Table Details of the output obtained from the MIPE and PrimerProspector analyses of the “Metagenome test set” with the primers F515 and R806. (XLS)

S4 Table Summary files F515.stat and R806.stat obtained from the MIPE analysis of the “Metatranscriptomic dataset SRX155355”. (XLS)

S1 File Krnoa chart of the MG-RAST SSU results obtained using MG-RAST dataset 4467420.3. (HTML)

S2 File Krnoa chart of SILVA SSU v119 results obtained using MG-RAST dataset 4467420.3. (HTML)

## References

[1] Pace NR. Mapping the tree of life: progress and prospects. Microbiol Mol Biol Rev. 2009;73:565–576. 10.1128/MMBR.00033-09 19946133 PMC2786576

[2] Woese CR. Bacterial evolution. Microbiology. 1987;51(2):221–271. 10.1128/mr.51.2.221-271.1987 PMC373105 2439888

[3] Woese CR. Default taxonomy: Ernst Mayr’s view of the microbial world. Proc Natl Acad Sci. 1998;95(19):11043–11046. 9736686 10.1073/pnas.95.19.11043 PMC33896

[4] Klindworth A, Pruesse E, Schweer T, Peplies J, Quast C, Horn M, et al Evaluation of general 16S ribosomal RNA gene PCR primers for classical and next-generation sequencing-based diversity studies. Nucleic Acids Res. 2013;41(1):e1 10.1093/nar/gks808 22933715 PMC3592464

[5] Cole JR, Wang Q, Fish JA, Chai B, McGarrell DM, Sun Y, et al Ribosomal Database Project: data and tools for high throughput rRNA analysis. Nucleic Acids Res. 2014;42(D1):D633–D642. 24288368 10.1093/nar/gkt1244 PMC3965039

[6] Johnson M, Zaretskaya I, Raytselis Y, Merezhuk Y, McGinnis S, Madden TL. NCBI BLAST: a better web interface. Nucleic Acids Res. 2008;36:W5–W9. 10.1093/nar/gkn201 18440982 PMC2447716

[7] DeSantis TZ, Hugenholtz P, Larsen N, Rojas M, Brodie EL, Keller K, et al Greengenes, a chimera-checked 16S rRNA gene database and workbench compatible with ARB. Appl Environ Microbiol. 2006;72(7):5069–5072. 10.1128/AEM.03006-05 16820507 PMC1489311

[8] Quast C, Pruesse E, Yilmaz P, Gerken J, Schweer T, Yarza P, et al The SILVA ribosomal RNA gene database project: improved data processing and web-based tools. Nucleic Acids Res. 2013;41:D590–D596. 10.1093/nar/gks1219 23193283 PMC3531112

[9] Greuter D, Loy A, Horn M, Rattei T. probeBase-an online resource for rRNA-targeted oligonucleotide probes and primers: new features 2016. Nucleic Acids Res. 2016;44(D1):D586–D589. 10.1093/nar/gkv1232 26586809 PMC4702872

[10] Mao D-P, Zhou Q, Chen C-Y, Quan Z-X. Coverage evaluation of universal bacterial primers using the metagenomic datasets. BMC Microbiol. 2012;12:66 10.1186/1471-2180-12-66 22554309 PMC3445835

[11] Rondon MR, August PR, Bettermann AD, Brady SF, Grossman TH, Liles MR, et al Cloning the soil metagenome: a strategy for accessing the genetic and functional diversity of uncultured microorganisms. Appl Environ Microbiol. 2000;66(6):2541–2547. 10831436 10.1128/aem.66.6.2541-2547.2000 PMC110579

[12] Goff SA, Vaughn M, McKay S, Lyons E, Stapleton AE, Gessler D, et al The iPlant collaborative: cyberinfrastructure for plant biology. Front Plant Sci. 2011;2:34 10.3389/fpls.2011.00034 22645531 PMC3355756

[13] Hunter S, Corbett M, Denise H, Fraser M, Gonzalez-Beltran A, Hunter C, et al EBI metagenomics—a new resource for the analysis and archiving of metagenomic data. Nucleic Acids Res. 2014;42(D1):D600–D606. 24165880 10.1093/nar/gkt961 PMC3965009

[14] Seshadri R, Kravitz S a., Smarr L, Gilna P, Frazier M. CAMERA: a community resource for metagenomics. Plos Biol. 2007;5(3):e75 10.1371/journal.pbio.0050075 17355175 PMC1821059

[15] Meyer F, Paarmann D, D’Souza M, Olson R, Glass EEM, Kubal M, et al The metagenomics RAST server–a public resource for the automatic phylogenetic and functional analysis of metagenomes. BMC Bioinformatics. 2008;9(1):386. 18803844 10.1186/1471-2105-9-386 PMC2563014

[16] Guo J, Cole JR, Zhang Q, Brown CT, Tiedje JM. Microbial community analysis with ribosomal gene fragments from shotgun metagenomes. Appl Environ Microbiol. 2015;82:157–166. 10.1128/AEM.02772-15 26475107 PMC4702641

[17] Sanschagrin S, Yergeau E. Next-generation Sequencing of 16S Ribosomal RNA Gene Amplicons. J Vis Exp. 2014;(90):51709. 10.3791/51709 PMC4828026 25226019

[18] Schloss PD, Westcott SL, Ryabin T, Hall JR, Hartmann M, Hollister EB, et al Introducing mothur: open-source, platform-independent, community-supported software for describing and comparing microbial communities. Appl Environ Microbiol. 2009;75(23):7537–7541. 10.1128/AEM.01541-09 19801464 PMC2786419

[19] Edgar RC. Search and clustering orders of magnitude faster than BLAST. Bioinformatics. 2010;26(19):2460–2461. 10.1093/bioinformatics/btq461 20709691

[20] Caporaso JG, Kuczynski J, Stombaugh J, Bittinger K, Bushman FD, Costello EK, et al QIIME allows analysis of high-throughput community sequencing data. Nat Methods. 2010;7(5):335–336. 10.1038/nmeth.f.303 20383131 PMC3156573

[21] Walker AW, Martin JC, Scott P, Parkhill J, Flint HJ, Scott KP. 16S rRNA gene-based profiling of the human infant gut microbiota is strongly influenced by sample processing and PCR primer choice. Microbiome. 2015;3:26 10.1186/s40168-015-0087-4 26120470 PMC4482049

[22] Miller CS, Baker BJ, Thomas BC, Singer SW, Banfield JF. EMIRGE: reconstruction of full-length ribosomal genes from microbial community short read sequencing data. Genome Biol. 2011;12(5):R44 10.1186/gb-2011-12-5-r44 21595876 PMC3219967

[23] Wilke A, Bischof J, Harrison T, Brettin T, D’Souza M, Gerlach W, et al A RESTful API for accessing microbial community data for MG-RAST. PLoS Comput Biol. 2015;11(1):e1004008 10.1371/journal.pcbi.1004008 25569221 PMC4287624

[24] Markowitz VM. The integrated microbial genomes (IMG) system. Nucleic Acids Res. 2006;34(90001):D344–D348. 16381883 10.1093/nar/gkj024 PMC1347387

[25] Walters WA, Caporaso JG, Lauber CL, Berg-Lyons D, Fierer N, Knight R. PrimerProspector: de novo design and taxonomic analysis of barcoded polymerase chain reaction primers. Bioinformatics. 2011;27(8):1159–1161. 10.1093/bioinformatics/btr087 21349862 PMC3072552

[26] Hugerth LW, Wefer HA, Lundin S, Jakobsson HE, Lindberg M, Rodin S, et al DegePrime, a program for degenerate primer design for broad-taxonomic-range PCR in microbial ecology studies. Appl Environ Microbiol. 2014;80(16):5116–5123. 10.1128/AEM.01403-14 24928874 PMC4135748

[27] Gulvik C a, Effler TC, Wilhelm SW, Buchan A. De-MetaST-BLAST: a tool for the validation of degenerate primer sets and data mining of publicly available metagenomes. PLoS One. 2012;7(11):e50362 10.1371/journal.pone.0050362 23189198 PMC3506598

[28] Li X-R, Lv Y, Meng H, Gu J-D, Quan Z-X. Analysis of microbial diversity by pyrosequencing the small-subunit ribosomal RNA without PCR amplification. Appl Microbiol Biotechnol. 2014;98(8):3777–3789. 10.1007/s00253-014-5583-0 24531274

[29] Biers EJ, Sun S, Howard EC. Prokaryotic genomes and diversity in surface ocean waters: interrogating the global ocean sampling metagenome. Appl Environ Microbiol. 2009;75(7):2221–2229. 10.1128/AEM.02118-08 19201952 PMC2663191

[30] Mou X, Sun S, Edwards RA, Hodson RE, Moran MA. Bacterial carbon processing by generalist species in the coastal ocean. Nature. 2008;451(7179):708–711. 10.1038/nature06513 18223640

[31] Bengtsson J, Eriksson KM, Hartmann M, Wang Z, Shenoy BD, Grelet GA, et al Metaxa: A software tool for automated detection and discrimination among ribosomal small subunit (12S/16S/18S) sequences of archaea, bacteria, eukaryotes, mitochondria, and chloroplasts in metagenomes and environmental sequencing datasets. Antonie van Leeuwenhoek, Int J Gen Mol Microbiol. 2011;100(3):471–475. 10.1007/s10482-011-9598-6 21674231

[32] Huang Y, Gilna P, Li W. Identification of ribosomal RNA genes in metagenomic fragments. Bioinformatics. 2009;25(10):1338–1340. 10.1093/bioinformatics/btp161 19346323 PMC2677747

[33] Kent WJ. BLAT—The BLAST-Like Alignment Tool. Genome Res. Cold Spring Harbor Laboratory Press; 2002 4 19;12(4):656–664. 10.1101/gr.229202 11932250 PMC187518

[34] Claesson MJ, O’Sullivan O, Wang Q, Nikkila J, Marchesi JR, Smidt H, et al Comparative analysis of pyrosequencing and a phylogenetic microarray for exploring microbial community structures in the human distal intestine. PLoS One. 2009;4(8):e6669 10.1371/journal.pone.0006669 19693277 PMC2725325

[35] Bru D, Martin-Laurent F, Philippot L. Quantification of the detrimental effect of a single primer-template mismatch by real-time PCR using the 16S rRNA gene as an example. Appl Environ Microbiol. 2008;74(5):1660–1663. 10.1128/AEM.02403-07 18192413 PMC2258636

[36] Wu JH, Hong PY, Liu WT. Quantitative effects of position and type of single mismatch on single base primer extension. J Microbiol Methods. 2009;77:267–275. 10.1016/j.mimet.2009.03.001 19285527

[37] Richter DC, Ott F, Auch AF, Schmid R, Huson DH. MetaSim—A sequencing simulator for genomics and metagenomics. PLoS One. 2008;3:10. 10.1371/journal.pone.0003373 PMC2556396 18841204

[38] Peabody MA, Van Rossum T, Lo R, Brinkman FSL. Evaluation of shotgun metagenomics sequence classification methods using in silico and in vitro simulated communities. BMC Bioinformatics. 2015;16:363. 10.1186/s12859-015-0788-5 PMC4634789 26537885

[39] Yu K, Zhang T. Metagenomic and metatranscriptomic analysis of microbial community structure and gene expression of activated sludge. PLoS One. 2012;7(5):e38183 10.1371/journal.pone.0038183 22666477 PMC3364235

[40] Bates ST, Berg-Lyons D, Caporaso JG, Walters WA, Knight R, Fierer N. Examining the global distribution of dominant archaeal populations in soil. ISME J. 2011;5(5):908–917. 10.1038/ismej.2010.171 21085198 PMC3105767

[41] Caporaso JG, Lauber CL, Walters WA, Berg-Lyons D, Lozupone CA, Turnbaugh PJ, et al Global patterns of 16S rRNA diversity at a depth of millions of sequences per sample. Proc Natl Acad Sci. 2011;108:4516–4522. 10.1073/pnas.1000080107 20534432 PMC3063599

[42] Ondov BD, Bergman NH, Phillippy AM. Interactive metagenomic visualization in a Web browser. BMC Bioinformatics. 2011;12(1):385. 21961884 10.1186/1471-2105-12-385 PMC3190407

