# Supplementary material for: MIPE: A metagenome-based community structure explorer and SSU primer evaluation tool
Source: PLoS One. 2017 Mar 28;12(3):e0174609. doi: 10.1371/journal.pone.0174609 (PMC5370157; doi:10.1371/journal.pone.0174609)
Supplement: S1 File — (HTML) [file pone.0174609.s005.html]

Javascript must be enabled to view this page.

magnitude
 12819
 2775
 2768
 738
 738
 738
 322
 316
 1
 4
 1
 3
 2
 1
 51
 50
 1
 362
 362
 2030
 1
 1
 1
 1
 196
 8
 8
 1
 7
 159
 159
 5
 46
 89
 19
 3
 3
 3
 26
 26
 15
 11
 1833
 975
 975
 59
 916
 858
 5
 5
 853
 35
 2
 789
 7
 1
 19
 7
 7
 7
 7
 7
 7
 8327
 46
 1
 1
 1
 1
 1
 32
 32
 32
 32
 32
 13
 13
 13
 10
 10
 3
 3
 2522
 2522
 5
 5
 4
 4
 1
 1
 2467
 5
 5
 5
 19
 2
 2
 13
 2
 1
 4
 2
 1
 2
 1
 4
 4
 4
 4
 4
 12
 12
 1
 1
 1
 3
 6
 3
 3
 2
 1
 21
 12
 12
 8
 2
 2
 2
 1
 1
 1
 1
 69
 69
 3
 31
 1
 1
 1
 8
 3
 1
 5
 3
 1
 1
 6
 1
 1
 2
 14
 14
 7
 7
 23
 1
 1
 22
 22
 8
 8
 8
 1
 1
 1
 33
 33
 23
 1
 3
 1
 3
 2
 18
 18
 18
 2
 2
 2
 288
 288
 1
 2
 99
 43
 6
 44
 2
 9
 8
 13
 1
 26
 1
 1
 2
 30
 409
 4
 4
 67
 10
 22
 27
 8
 3
 3
 84
 1
 3
 80
 1
 1
 250
 89
 41
 57
 27
 36
 1
 1
 1
 6
 6
 6
 155
 5
 5
 3
 1
 2
 1
 1
 1
 1
 25
 25
 10
 8
 2
 3
 3
 31
 5
 5
 21
 1
 1
 64
 1
 1
 3
 20
 2
 1
 1
 1
 2
 3
 1
 15
 6
 1
 1
 2
 2
 1
 1
 1
 8
 8
 2
 2
 135
 82
 5
 34
 10
 2
 1
 6
 2
 1
 1
 6
 12
 1
 1
 5
 1
 3
 1
 26
 22
 3
 1
 1
 1
 21
 17
 3
 1
 31
 4
 1
 3
 3
 3
 3
 2
 1
 13
 8
 1
 4
 8
 2
 5
 1
 561
 561
 1
 1
 16
 1
 1
 26
 1
 1
 1
 3
 16
 11
 2
 2
 3
 1
 3
 1
 1
 11
 2
 42
 3
 2
 2
 4
 1
 4
 26
 9
 16
 2
 8
 3
 3
 8
 48
 6
 26
 40
 3
 5
 3
 2
 6
 22
 2
 21
 1
 2
 1
 1
 3
 3
 9
 34
 1
 5
 1
 9
 1
 1
 6
 1
 5
 3
 2
 9
 32
 8
 1
 14
 14
 14
 167
 48
 14
 1
 1
 2
 4
 2
 3
 7
 3
 11
 118
 3
 1
 19
 21
 3
 2
 2
 26
 1
 12
 9
 7
 7
 1
 1
 1
 2
 1
 1
 110
 15
 15
 12
 2
 7
 1
 2
 80
 35
 4
 11
 4
 1
 1
 7
 2
 3
 1
 7
 4
 3
 3
 27
 7
 3
 1
 3
 20
 1
 19
 18
 14
 14
 2
 2
 2
 2
 70
 24
 24
 45
 3
 5
 8
 16
 10
 1
 1
 1
 1
 1
 41
 3
 3
 11
 1
 8
 1
 1
 1
 1
 2
 2
 6
 6
 16
 3
 2
 1
 3
 4
 1
 1
 1
 1
 1
 1
 1
 3
 3
 3
 6
 6
 6
 11
 11
 11
 93
 32
 32
 2
 2
 59
 2
 7
 1
 5
 1
 2
 1
 2
 2
 1
 8
 4
 2
 3
 1
 1
 1
 1
 1
 1
 2
 4
 2
 2
 2
 11
 1
 1
 1
 1
 4
 4
 5
 2
 3
 11
 3
 1
 2
 4
 1
 1
 2
 1
 1
 3
 3
 48
 48
 28
 17
 3
 9
 9
 9
 10
 10
 10
 32
 32
 31
 2
 9
 3
 4
 1
 6
 1
 4
 1
 1
 1
 2
 2
 1
 1
 1
 1
 11
 11
 11
 11
 1
 1
 1
 1
 4
 4
 4
 3
 1
 897
 60
 60
 15
 15
 1
 2
 3
 2
 6
 1
 19
 2
 2
 5
 1
 3
 1
 12
 2
 2
 2
 1
 2
 3
 6
 2
 2
 4
 2
 2
 18
 16
 15
 1
 2
 2
 2
 2
 2
 160
 160
 7
 7
 7
 138
 87
 2
 15
 12
 4
 2
 23
 1
 23
 4
 1
 4
 4
 2
 2
 21
 5
 14
 2
 2
 1
 1
 3
 3
 18
 18
 1
 1
 15
 8
 8
 4
 4
 3
 3
 427
 424
 423
 5
 5
 109
 29
 67
 2
 3
 8
 5
 1
 3
 1
 36
 1
 4
 3
 2
 6
 5
 2
 9
 4
 11
 11
 9
 9
 4
 4
 156
 29
 1
 1
 3
 5
 61
 27
 14
 4
 1
 3
 7
 6
 6
 6
 2
 4
 2
 2
 19
 16
 3
 1
 1
 1
 1
 4
 4
 34
 33
 1
 13
 2
 3
 8
 2
 1
 1
 1
 1
 1
 3
 3
 3
 3
 242
 242
 2
 2
 2
 34
 15
 7
 1
 1
 1
 5
 16
 2
 1
 13
 3
 3
 206
 90
 59
 31
 116
 78
 38
 8
 3
 3
 1
 1
 2
 2
 5
 5
 1
 1
 4
 4
 14
 14
 14
 7
 1
 1
 6
 1
 1
 1
 1
 1
 1
 7
 5
 5
 2
 2
 5
 5
 5
 5
 1
 1
 4
 4
 72
 46
 35
 26
 8
 2
 6
 18
 17
 1
 9
 9
 9
 11
 11
 11
 11
 12
 12
 12
 12
 4
 4
 4
 1
 1
 1
 1
 1
 13
 9
 9
 9
 9
 4
 4
 4
 4
 37
 5
 5
 5
 5
 5
 32
 13
 13
 6
 6
 7
 1
 3
 3
 15
 15
 14
 2
 12
 1
 1
 4
 4
 1
 1
 2
 1
 1
 1
 1
 1
 1
 1
 1
 1
 1
 15
 15
 2
 2
 2
 1
 1
 13
 13
 4
 3
 1
 9
 5
 4
 4
 4
 4
 4
 4
 4
 483
 312
 69
 32
 29
 3
 2
 1
 1
 1
 1
 16
 4
 1
 1
 1
 1
 1
 1
 26
 7
 1
 6
 19
 1
 17
 1
 1
 1
 1
 8
 2
 2
 6
 4
 1
 1
 1
 1
 1
 1
 1
 1
 243
 3
 2
 2
 1
 1
 3
 2
 2
 1
 1
 4
 3
 1
 2
 1
 1
 161
 151
 1
 3
 2
 14
 5
 1
 5
 6
 9
 4
 3
 1
 77
 1
 1
 6
 1
 2
 2
 7
 10
 10
 16
 5
 2
 1
 1
 1
 11
 1
 10
 56
 23
 1
 2
 10
 8
 2
 33
 3
 1
 1
 7
 2
 5
 3
 1
 5
 5
 150
 137
 38
 1
 1
 35
 1
 2
 3
 3
 1
 1
 1
 1
 1
 4
 1
 1
 2
 1
 2
 1
 3
 1
 2
 3
 1
 1
 1
 1
 5
 1
 1
 4
 1
 1
 2
 1
 1
 1
 4
 4
 1
 1
 1
 1
 4
 4
 1
 3
 8
 3
 3
 1
 1
 1
 1
 2
 2
 1
 1
 14
 6
 2
 4
 7
 1
 1
 2
 2
 1
 1
 1
 42
 1
 1
 41
 2
 7
 8
 4
 10
 1
 9
 16
 1
 1
 4
 4
 5
 2
 1
 2
 6
 6
 5
 2
 2
 3
 1
 1
 1
 3
 3
 3
 3
 10
 8
 1
 1
 1
 1
 6
 1
 1
 1
 3
 1
 1
 1
 1
 1
 1
 3
 3
 3
 3
 3
 18
 18
 1
 1
 1
 17
 3
 2
 1
 3
 3
 4
 1
 1
 2
 3
 3
 2
 2
 2
 1
 1
 10
 10
 10
 8
 3
 2
 1
 5
 5
 2
 2
 2
 6
 6
 6
 6
 6
 6
 1
 1
 1
 1
 1
 1
 135
 135
 135
 135
 1
 1
 134
 134
 110
 110
 110
 110
 3
 3
 12
 12
 20
 20
 12
 12
 40
 24
 16
 23
 9
 1
 9
 1
 2
 1
 3631
 1073
 33
 33
 2
 2
 13
 9
 2
 1
 1
 13
 2
 1
 3
 7
 5
 3
 2
 3
 3
 3
 3
 590
 1
 1
 1
 53
 53
 4
 1
 1
 1
 1
 44
 1
 32
 21
 21
 1
 1
 9
 8
 1
 1
 1
 167
 1
 1
 10
 1
 5
 1
 1
 2
 119
 23
 7
 7
 2
 5
 1
 22
 13
 13
 13
 13
 2
 2
 18
 18
 17
 1
 16
 17
 10
 2
 6
 1
 1
 7
 5
 2
 31
 7
 3
 4
 24
 3
 12
 1
 2
 3
 2
 1
 34
 34
 1
 2
 1
 5
 19
 1
 4
 1
 153
 1
 1
 78
 3
 2
 45
 2
 3
 2
 5
 1
 1
 6
 1
 5
 2
 2
 2
 69
 1
 1
 30
 37
 3
 2
 1
 31
 9
 6
 3
 1
 1
 16
 1
 3
 2
 2
 3
 5
 1
 1
 4
 4
 58
 23
 17
 6
 7
 7
 27
 5
 2
 1
 16
 1
 2
 1
 1
 2
 2
 2
 10
 1
 1
 9
 7
 2
 1
 1
 1
 186
 20
 2
 2
 3
 3
 15
 15
 165
 2
 2
 1
 1
 1
 1
 4
 4
 54
 2
 2
 8
 1
 35
 1
 2
 1
 2
 7
 7
 17
 17
 3
 3
 1
 1
 42
 2
 11
 2
 1
 7
 19
 15
 5
 1
 4
 5
 5
 1
 4
 5
 1
 1
 3
 1
 1
 1
 1
 2
 1
 1
 3
 3
 1
 1
 1
 1
 1
 134
 106
 64
 1
 1
 61
 1
 10
 5
 5
 3
 3
 7
 2
 3
 1
 1
 10
 10
 1
 1
 1
 1
 5
 5
 5
 5
 28
 18
 12
 4
 1
 1
 1
 1
 6
 3
 3
 3
 3
 24
 2
 1
 1
 1
 1
 21
 21
 19
 2
 1
 1
 1
 95
 2
 1
 1
 1
 1
 93
 3
 3
 29
 12
 4
 2
 2
 9
 1
 1
 20
 2
 1
 3
 12
 2
 33
 3
 5
 11
 3
 3
 1
 2
 1
 1
 2
 1
 2
 1
 1
 2
 2
 3
 3
 8
 8
 3
 3
 5
 4
 1
 1323
 815
 67
 2
 1
 1
 12
 10
 2
 12
 3
 9
 9
 7
 1
 1
 3
 3
 13
 13
 6
 6
 4
 4
 6
 2
 4
 162
 85
 8
 10
 2
 5
 17
 2
 4
 1
 1
 5
 2
 2
 1
 1
 1
 1
 4
 3
 1
 2
 3
 4
 3
 2
 1
 1
 13
 3
 4
 1
 1
 4
 1
 1
 11
 1
 2
 8
 2
 2
 7
 3
 1
 3
 42
 8
 5
 3
 6
 2
 6
 1
 4
 6
 1
 346
 135
 17
 2
 49
 3
 1
 8
 38
 4
 10
 1
 1
 1
 25
 25
 18
 14
 4
 4
 4
 48
 25
 6
 1
 9
 1
 4
 2
 22
 22
 10
 10
 12
 9
 3
 1
 1
 14
 4
 1
 9
 2
 1
 1
 3
 1
 2
 9
 9
 1
 1
 28
 8
 2
 3
 13
 2
 5
 5
 8
 8
 1
 1
 61
 1
 1
 1
 1
 23
 2
 1
 1
 1
 2
 2
 2
 2
 2
 2
 2
 2
 2
 9
 1
 7
 1
 9
 3
 6
 7
 7
 8
 2
 6
 2
 2
 1
 1
 179
 29
 1
 22
 6
 3
 3
 2
 2
 4
 4
 1
 1
 15
 15
 42
 39
 3
 42
 42
 6
 6
 11
 11
 17
 4
 2
 1
 3
 7
 7
 7
 5
 5
 5
 5
 25
 25
 25
 2
 9
 7
 3
 4
 25
 25
 21
 21
 3
 2
 1
 1
 1
 70
 70
 1
 1
 3
 3
 3
 3
 9
 2
 1
 6
 1
 1
 1
 1
 11
 11
 1
 1
 35
 2
 4
 1
 11
 14
 2
 1
 1
 1
 1
 1
 3
 3
 47
 47
 24
 1
 4
 1
 4
 4
 2
 1
 3
 4
 18
 7
 2
 6
 1
 2
 4
 4
 1
 1
 316
 316
 3
 3
 35
 1
 3
 13
 1
 5
 12
 11
 10
 1
 44
 15
 29
 14
 14
 73
 5
 17
 4
 2
 1
 6
 12
 5
 17
 4
 7
 2
 5
 18
 18
 4
 4
 4
 4
 32
 5
 1
 1
 1
 1
 6
 11
 2
 2
 2
 70
 10
 37
 23
 1
 1
 20
 20
 1
 1
 7
 7
 10
 10
 2
 2
 271
 2
 1
 1
 1
 1
 1
 1
 17
 6
 2
 1
 1
 1
 1
 1
 1
 1
 1
 1
 1
 11
 6
 6
 5
 5
 41
 4
 4
 4
 2
 2
 1
 1
 22
 1
 1
 20
 3
 2
 7
 1
 2
 1
 3
 1
 1
 1
 13
 13
 13
 2
 2
 2
 2
 22
 5
 2
 1
 1
 3
 3
 15
 15
 1
 10
 1
 1
 1
 1
 2
 2
 2
 175
 5
 4
 2
 2
 1
 1
 22
 22
 16
 6
 77
 52
 46
 3
 3
 9
 2
 7
 16
 5
 1
 10
 22
 9
 9
 13
 13
 46
 9
 9
 37
 37
 3
 3
 3
 10
 2
 2
 2
 8
 7
 7
 1
 1
 2
 2
 2
 1
 1
 34
 28
 20
 8
 2
 3
 3
 7
 4
 2
 1
 5
 1
 4
 8
 6
 1
 1
 4
 2
 2
 1
 1
 1
 1
 5
 5
 5
 5
 913
 9
 9
 9
 1
 7
 1
 15
 14
 10
 1
 1
 3
 5
 4
 4
 1
 1
 1
 17
 6
 1
 1
 3
 3
 2
 2
 1
 1
 1
 2
 2
 1
 1
 8
 8
 3
 1
 1
 1
 2
 4
 4
 4
 4
 30
 14
 1
 1
 1
 1
 7
 3
 1
 3
 1
 1
 2
 2
 2
 2
 10
 8
 8
 2
 2
 6
 4
 1
 2
 1
 2
 2
 49
 49
 6
 6
 2
 2
 1
 1
 1
 1
 5
 1
 4
 16
 11
 5
 1
 1
 7
 3
 3
 1
 2
 2
 1
 1
 1
 1
 3
 3
 2
 1
 1
 1
 1
 225
 210
 210
 209
 1
 15
 15
 2
 1
 1
 2
 1
 5
 1
 1
 1
 4
 4
 1
 1
 2
 2
 1
 1
 25
 6
 6
 1
 1
 2
 2
 8
 8
 8
 5
 2
 2
 3
 3
 6
 5
 2
 2
 1
 1
 1
 5
 5
 1
 1
 1
 1
 1
 1
 2
 1
 1
 132
 106
 77
 8
 1
 13
 1
 6
 10
 4
 1
 8
 2
 3
 1
 17
 1
 1
 23
 1
 1
 21
 6
 5
 1
 26
 26
 2
 1
 5
 1
 1
 3
 1
 2
 1
 6
 2
 1
 9
 4
 4
 2
 2
 5
 2
 1
 1
 3
 1
 1
 1
 8
 8
 2
 2
 1
 1
 5
 3
 2
 353
 353
 9
 9
 10
 9
 1
 37
 18
 19
 21
 21
 1
 1
 136
 4
 126
 4
 2
 112
 58
 5
 21
 2
 5
 21
 27
 27
 28
 28
 1
 1
 5
 5
 2
 2
 20
 1
 2
 7
 1
 1
 1
 6
 1
 17
 17
 17
 17
 1
 16
 16
 16
 16
 4
 4
 4
 2
 2
 1
 1
 10
 1
 1
 5
 2
 3
 4
 2
 1
 1
 9
 9
 9
 9
 1
 1
 5
 5
 2
 2
 1
 1
 15
 15
 3
 3
 2
 1
 1
 1
 1
 5
 3
 3
 3
 2
 2
 1
 1
 7
 7
 7
 1
 1
 2
 1
 2
 2
 2
 2
 2
 2
 2
 101
 22
 1
 1
 1
 1
 21
 21
 3
 3
 18
 5
 13
 4
 4
 4
 4
 4
 64
 64
 39
 39
 39
 25
 4
 4
 8
 1
 1
 6
 1
 1
 8
 8
 4
 4
 11
 6
 6
 6
 4
 2
 5
 5
 5
 5
 195
 195
 195
 195
 7
 7
 2
 2
 186
 6
 10
 28
 8
 2
 4
 16
 1
 4
 1
 12
 19
 1
 6
 10
 9
 22
 27
 1714
 67
 1
 1
 1
 1
 1
 4
 1
 1
 1
 1
 3
 3
 3
 3
 62
 62
 59
 1
 1
 55
 55
 1
 1
 2
 1
 1
 3
 2
 2
 1
 1
 4
 1
 1
 1
 1
 1
 3
 3
 2
 2
 2
 1
 1
 1
 124
 3
 1
 1
 1
 1
 1
 1
 1
 1
 1
 1
 1
 1
 1
 1
 1
 1
 1
 109
 2
 1
 1
 1
 1
 1
 1
 3
 2
 1
 1
 1
 1
 1
 1
 1
 2
 1
 1
 1
 1
 1
 1
 5
 1
 1
 1
 2
 1
 1
 1
 1
 2
 2
 2
 94
 94
 94
 94
 1
 1
 1
 1
 2
 2
 2
 2
 1
 1
 1
 1
 1
 10
 1
 1
 1
 1
 2
 2
 2
 2
 7
 7
 7
 7
 256
 5
 1
 1
 1
 1
 1
 1
 1
 1
 3
 1
 1
 1
 2
 2
 2
 112
 7
 7
 3
 3
 4
 4
 105
 104
 104
 1
 103
 1
 1
 1
 18
 14
 1
 1
 1
 13
 13
 11
 2
 4
 3
 2
 2
 1
 1
 1
 1
 1
 2
 2
 2
 2
 2
 2
 2
 2
 2
 2
 22
 22
 1
 1
 1
 1
 1
 1
 1
 1
 1
 2
 2
 1
 1
 8
 1
 1
 1
 1
 5
 5
 1
 1
 9
 9
 1
 1
 2
 1
 1
 1
 1
 1
 95
 94
 94
 94
 89
 5
 1
 1
 1
 1
 7
 3
 1
 1
 1
 1
 2
 1
 1
 1
 1
 1
 1
 3
 1
 1
 1
 1
 2
 2
 2
 1
 1
 1
 1
 1
 1
 1
 5
 1
 1
 1
 1
 1
 2
 2
 2
 2
 1
 1
 2
 2
 2
 1
 1
 1
 1
 1
 1
 1
 1
 1
 1
 19
 4
 1
 1
 1
 1
 3
 3
 3
 3
 4
 1
 1
 1
 1
 2
 2
 1
 1
 1
 1
 1
 1
 1
 1
 9
 7
 7
 1
 1
 6
 5
 1
 1
 1
 1
 1
 1
 1
 1
 1
 2
 2
 2
 2
 2
 799
 796
 796
 796
 796
 796
 2
 2
 2
 2
 2
 1
 1
 1
 1
 1
 2
 1
 1
 1
 1
 1
 1
 1
 1
 1
 1
 17
 3
 3
 2
 2
 2
 1
 1
 1
 14
 14
 14
 14
 14
 4
 4
 3
 2
 2
 2
 1
 1
 1
 1
 1
 1
 1
 10
 1
 1
 1
 1
 1
 9
 1
 1
 1
 1
 8
 8
 1
 1
 7
 4
 3
 1
 1
 1
 1
 1
 1
 3
 3
 1
 1
 1
 1
 2
 2
 2
 2
 3
 3
 1
 1
 1
 1
 2
 1
 1
 1
 1
 1
 1
 1
 1
 1
 1
 1
 1
 4
 4
 2
 2
 2
 2
 2
 2
 2
 2
 3
 2
 2
 2
 2
 2
 1
 1
 1
 1
 1
 1
 1
 1
 1
 1
 1
 20
 1
 1
 1
 1
 1
 19
 4
 3
 1
 1
 2
 2
 1
 1
 1
 15
 1
 1
 1
 1
 1
 1
 3
 3
 2
 1
 2
 2
 2
 7
 5
 5
 1
 1
 1
 1
 1
 1
 1
 1
 1
 1
 1
 1
 1
 78
 5
 5
 5
 5
 5
 11
 11
 11
 11
 11
 1
 1
 1
 1
 1
 34
 1
 1
 1
 1
 33
 33
 2
 2
 22
 22
 9
 9
 4
 4
 4
 4
 4
 1
 1
 1
 1
 1
 6
 6
 6
 6
 1
 1
 1
 1
 1
 1
 1
 1
 1
 1
 1
 15
 5
 5
 1
 1
 4
 4
 1
 1
 1
 1
 1
 1
 1
 1
 1
 1
 1
 1
 2
 2
 2
 2
 1
 1
 1
 1
 4
 4
 4
 4
 52
 52
 1
 1
 1
 1
 51
 40
 1
 1
 1
 1
 1
 1
 10
 10
 1
 1
 19
 1
 8
 9
 1
 4
 4
 3
 3
 11
 6
 6
 5
 5
 5
 5
 2
 2
 2
 2
 3
 3
 3
 3
 221
 1
 1
 1
 1
 1
 1
 1
 1
 1
 1
 3
 3
 2
 2
 1
 1
 1
 1
 1
 1
 1
 1
 1
 1
 7
 1
 1
 1
 1
 5
 5
 5
 5
 1
 1
 1
 1
 2
 2
 2
 1
 1
 1
 1
 6
 3
 3
 1
 1
 1
 1
 1
 1
 3
 3
 3
 3
 5
 2
 2
 2
 2
 1
 1
 1
 1
 2
 2
 2
 2
 1
 1
 1
 1
 1
 4
 4
 4
 4
 2
 1
 1
 2
 2
 2
 2
 2
 1
 1
 1
 1
 1
 7
 3
 3
 2
 2
 1
 1
 4
 4
 4
 4
 49
 1
 1
 1
 1
 2
 2
 2
 2
 2
 2
 2
 2
 2
 2
 2
 2
 42
 7
 7
 7
 8
 3
 3
 5
 5
 25
 1
 1
 24
 4
 11
 9
 2
 2
 2
 1
 1
 1
 1
 1
 4
 4
 4
 4
 4
 2
 2
 2
 2
 2
 1
 1
 1
 1
 1
 10
 1
 1
 1
 1
 6
 6
 6
 6
 3
 1
 1
 1
 2
 1
 1
 1
 1
 113
 10
 10
 10
 10
 18
 8
 7
 3
 1
 2
 1
 1
 1
 6
 3
 3
 3
 1
 2
 2
 2
 2
 2
 2
 2
 4
 1
 1
 1
 3
 3
 2
 1
 1
 1
 1
 1
 5
 4
 3
 1
 1
 1
 1
 1
 1
 1
 1
 3
 3
 3
 3
 2
 2
 2
 1
 1
 2
 1
 1
 1
 1
 1
 1
 1
 1
 1
 1
 2
 2
 2
 2
 1
 1
 1
 1
 21
 5
 4
 4
 1
 1
 16
 16
 11
 2
 3
 1
 1
 1
 1
 1
 1
 1
 1
 7
 7
 6
 6
 1
 1
 2
 2
 1
 1
 1
 1
 32
 1
 1
 1
 1
 1
 1
 2
 2
 2
 6
 1
 1
 5
 5
 1
 1
 1
 2
 2
 2
 1
 1
 1
 1
 1
 1
 17
 1
 1
 4
 4
 3
 3
 2
 2
 1
 1
 1
 1
 1
 1
 1
 1
 3
 3
 6
 6
 1
 1
 1
 1
 2
 2
 2
 1
 1
 1
 1
 1
 1
 2
 1
 1
 1
 1
 1
 1
 3
 3
 3
 2
 1
 1
 1
 1
 1
 1
 1
 1
 1
 1
