# Supplementary material for: MIPE: A metagenome-based community structure explorer and SSU primer evaluation tool
Source: PLoS One. 2017 Mar 28;12(3):e0174609. doi: 10.1371/journal.pone.0174609 (PMC5370157; doi:10.1371/journal.pone.0174609)
Supplement: S2 File — (HTML) [file pone.0174609.s006.html]

Javascript must be enabled to view this page.

magnitude
 12620
 3249
 1
 1
 1
 1
 1
 3061
 502
 502
 502
 6
 1
 493
 2
 2197
 2
 2
 2
 1
 1
 1
 276
 1
 1
 263
 104
 144
 15
 4
 3
 1
 8
 8
 1866
 3
 3
 52
 52
 1052
 1
 1011
 40
 759
 759
 52
 52
 52
 303
 281
 80
 80
 5
 5
 196
 196
 18
 18
 18
 4
 4
 4
 59
 59
 59
 59
 78
 78
 78
 78
 78
 109
 109
 109
 109
 109
 8886
 140
 97
 2
 2
 1
 1
 39
 11
 11
 28
 22
 2
 4
 31
 15
 15
 5
 5
 11
 11
 14
 14
 14
 11
 11
 11
 42
 42
 42
 35
 3
 4
 1
 1
 1
 1
 2051
 57
 57
 20
 7
 9
 4
 8
 8
 8
 8
 21
 21
 1899
 2
 2
 1
 1
 10
 10
 9
 1
 645
 1
 1
 205
 205
 306
 223
 19
 6
 56
 2
 35
 35
 98
 98
 39
 4
 1
 2
 1
 6
 3
 3
 13
 13
 16
 16
 9
 9
 2
 5
 2
 491
 7
 7
 9
 9
 1
 1
 2
 1
 1
 1
 1
 257
 42
 26
 9
 93
 87
 38
 1
 1
 2
 2
 18
 1
 13
 7
 4
 1
 2
 3
 1
 1
 1
 166
 166
 20
 20
 3
 17
 1
 1
 1
 155
 49
 1
 44
 4
 99
 7
 36
 1
 2
 4
 49
 7
 7
 7
 7
 1
 2
 1
 3
 25
 25
 1
 8
 16
 3
 2
 1
 1
 1
 1
 492
 492
 492
 38
 1
 1
 1
 36
 11
 11
 3
 3
 11
 11
 11
 11
 1
 1
 1
 57
 57
 57
 57
 9
 1
 1
 1
 1
 8
 8
 8
 8
 967
 7
 7
 5
 1
 1
 1
 2
 1
 1
 1
 1
 130
 128
 8
 5
 1
 2
 63
 7
 2
 1
 1
 1
 7
 1
 7
 36
 4
 1
 3
 2
 2
 51
 51
 2
 2
 2
 259
 259
 2
 1
 1
 246
 1
 6
 29
 30
 2
 2
 45
 2
 6
 4
 1
 1
 3
 1
 1
 6
 106
 5
 5
 6
 6
 447
 447
 363
 15
 73
 2
 13
 16
 1
 11
 15
 1
 1
 10
 205
 15
 15
 12
 12
 5
 5
 9
 1
 2
 1
 5
 2
 2
 37
 37
 4
 4
 121
 121
 121
 121
 2
 2
 2
 2
 1
 1
 1
 1
 4
 4
 4
 4
 4
 22
 22
 22
 22
 22
 4
 4
 4
 4
 4
 152
 148
 148
 148
 148
 4
 4
 4
 4
 7
 7
 7
 1
 1
 4
 2
 2
 2
 2
 6
 6
 6
 3
 3
 2
 2
 1
 1
 165
 12
 12
 12
 3
 1
 8
 4
 4
 4
 4
 24
 24
 24
 16
 8
 81
 81
 75
 75
 6
 6
 1
 1
 1
 1
 8
 6
 6
 6
 2
 2
 2
 35
 35
 35
 35
 2
 1
 1
 1
 1
 1
 1
 1
 1
 2
 2
 1
 1
 1
 1
 1
 1
 282
 188
 20
 13
 1
 12
 1
 1
 6
 6
 99
 4
 1
 3
 56
 35
 9
 12
 2
 2
 31
 7
 20
 4
 6
 6
 69
 69
 69
 56
 55
 9
 5
 1
 1
 2
 1
 1
 6
 1
 5
 1
 1
 23
 18
 5
 8
 1
 1
 2
 1
 1
 2
 7
 7
 1
 1
 1
 2
 2
 2
 1
 1
 5
 5
 5
 1
 1
 2
 1
 31
 31
 31
 31
 1
 1
 1
 1
 1
 6
 6
 5
 5
 5
 1
 1
 1
 126
 126
 126
 126
 126
 145
 3
 3
 3
 3
 14
 10
 10
 5
 4
 1
 2
 2
 2
 2
 2
 2
 111
 110
 110
 3
 2
 7
 8
 1
 36
 22
 31
 1
 1
 1
 17
 17
 17
 17
 3709
 1229
 44
 22
 9
 4
 5
 4
 20
 3
 1
 7
 9
 2
 2
 511
 2
 2
 1
 1
 2
 2
 2
 2
 14
 3
 4
 7
 99
 6
 11
 59
 2
 21
 46
 1
 8
 2
 16
 4
 3
 11
 1
 5
 2
 2
 1
 99
 67
 12
 20
 3
 3
 8
 5
 1
 1
 1
 5
 5
 26
 3
 2
 12
 9
 194
 194
 5
 1
 1
 1
 2
 216
 216
 50
 1
 1
 1
 7
 27
 6
 17
 2
 6
 98
 101
 28
 1
 6
 1
 3
 17
 1
 1
 10
 1
 1
 3
 5
 22
 18
 4
 40
 40
 4
 3
 3
 1
 1
 101
 16
 3
 9
 2
 2
 76
 13
 7
 1
 7
 13
 1
 33
 1
 9
 9
 252
 252
 252
 1067
 550
 15
 1
 5
 1
 2
 3
 3
 30
 4
 5
 5
 5
 3
 8
 405
 11
 14
 16
 2
 5
 1
 19
 4
 23
 1
 3
 1
 9
 3
 1
 5
 1
 1
 4
 37
 10
 8
 1
 220
 5
 4
 4
 96
 96
 11
 11
 2
 9
 4
 4
 3
 1
 23
 23
 1
 4
 18
 21
 19
 9
 5
 5
 2
 2
 198
 198
 1
 2
 3
 5
 40
 7
 4
 2
 4
 4
 16
 2
 61
 47
 37
 37
 37
 223
 223
 223
 265
 3
 2
 2
 1
 1
 1
 1
 1
 3
 1
 1
 2
 2
 1
 1
 1
 2
 2
 2
 167
 2
 2
 14
 4
 2
 8
 40
 40
 7
 3
 4
 23
 3
 13
 7
 13
 12
 1
 68
 68
 2
 2
 2
 1
 1
 1
 85
 85
 85
 6
 6
 6
 5
 1
 655
 1
 1
 1
 1
 1
 1
 11
 11
 8
 1
 2
 4
 4
 1
 3
 16
 16
 1
 2
 2
 11
 1
 1
 1
 2
 2
 1
 1
 5
 2
 1
 1
 3
 3
 1
 1
 1
 84
 73
 48
 20
 1
 4
 11
 9
 1
 1
 2
 1
 1
 1
 1
 142
 142
 142
 1
 1
 1
 384
 5
 4
 1
 7
 7
 367
 5
 9
 22
 30
 1
 5
 4
 42
 71
 155
 23
 5
 5
 1
 1
 1
 1
 486
 486
 486
 486
 16
 16
 16
 16
 16
 1
 1
 1
 1
 1
 3
 3
 3
 3
 3
 967
 967
 967
 967
 967
 97
 1
 1
 1
 1
 55
 49
 49
 49
 6
 6
 6
 7
 4
 4
 1
 2
 1
 1
 1
 1
 2
 2
 2
 4
 4
 1
 1
 3
 3
 20
 20
 20
 20
 10
 10
 10
 1
 9
 2
 2
 2
 2
 2
 485
 2
 1
 1
 1
 1
 1
 1
 1
 1
 13
 7
 2
 2
 2
 3
 1
 1
 2
 2
 2
 2
 2
 6
 6
 6
 6
 4
 3
 3
 3
 3
 1
 1
 1
 1
 198
 115
 115
 115
 47
 5
 1
 1
 1
 8
 1
 1
 50
 48
 42
 1
 1
 15
 15
 1
 1
 25
 25
 1
 1
 1
 5
 5
 5
 35
 35
 35
 35
 68
 29
 23
 23
 1
 6
 6
 8
 2
 2
 2
 2
 4
 4
 4
 20
 12
 1
 1
 2
 1
 1
 2
 2
 7
 7
 2
 2
 2
 6
 6
 6
 19
 19
 19
 19
 200
 200
 200
 200
 200
